# Internalizing Symptoms Among Youth in Foster Care: Prevalence and Associations with Exposure to Maltreatment

**DOI:** 10.1007/s10578-020-01118-x

**Published:** 2021-02-11

**Authors:** Yasmin Moussavi, Gro Janne Wergeland, Tormod Bøe, Bente Storm Mowatt Haugland, Marit Larsen, Stine Lehmann

**Affiliations:** 1 Regional Centre for Child and Youth Mental Health and Child Welfare - West, NORCE Norwegian Research Centre, P.O.B 22 Nygårdstangen, 5838 Bergen, Norway; 2 Department of Health Promotion and Development, Faculty of Psychology, University of Bergen, Bergen, Norway; 3 Department of Child and Adolescent Psychiatry, Division of Psychiatry, Haukeland University Hospital, Bergen, Norway; 4 Department of Clinical Medicine, Faculty of Medicine, University of Bergen, Bergen, Norway; 5 Department of Psychosocial Science, Faculty of Psychology, University of Bergen, Bergen, Norway; 6 Department of Clinical Psychology, Faculty of Psychology, University of Bergen, Bergen, Norway

**Keywords:** Youth in foster care, Anxiety symptoms, Depressive symptoms, Maltreatment, Sexual abuse

## Abstract

Among youth in foster care (*N* = 303, aged 11–17 years), we investigated prevalence of internalizing symptoms; associations between symptom level and maltreatment types and numbers; and the interaction between gender and maltreatment, on internalizing symptoms. Youth completed Spence Children Anxiety Scale, Short Mood Feelings Questionnaire, and Child and Adolescent Trauma Screen. Compared to community samples, symptom levels above clinical cut-off was more frequent, with social- and generalized anxiety (ES = 0.78–0.88) being most prevalent among youth in foster care. Girls reported more internalizing symptoms (ES = 0.59–0.93). Sexual abuse and neglect were associated with a broader range of internalizing symptoms (ES = 0.35–0.64). Increased incidence of maltreatment was associated with increased levels of symptoms (ES = 0.21–0.22). Associations between maltreatment and symptom level were stronger for girls. This study stresses the importance of broad screening of maltreatment and internalizing symptoms to meet the needs of youth in foster care.

## Introduction

Youth placed in foster and residential care show a heightened risk for depressive and anxiety disorders compared to the general youth population [1]. A point prevalence of 37.0% for depressive disorder and 34.0% for anxiety disorders has been found among youth placed in residential care [2]. A study among school-aged foster children has shown that 24.0% meet criteria for internalizing disorders [3]. However, for youth in foster care, more detailed reports of level and pattern of internalizing symptoms and impairment are scarce. Focusing on symptom level instead of diagnosis could provide important information as youth in foster care often show high, but sub-threshold scores on several symptom subscales simultaneously [4, 5]. Consequently, they run a higher risk of impairment [6] without meeting criteria for a diagnosis. This has important clinical implications for how the needs of youth in foster care are assessed and met in the Child Welfare Services (CWS) and the Child and Adolescent Mental Health Services (CAMHS).

While externalizing disorders demonstrate visible challenging behaviors frequently leading to assessment and interventions, the more invisible internalizing symptoms may go undetected and consequently without necessary follow-up. The diverse interventions available for youth in foster care with externalizing disorders (e.g. Multisystemic Therapy, Aggression Replacement Training and Treatment Foster Care Oregon [7]) may reflect this predominant focus. Consequently, a range of well-developed interventions are available for youth in foster care having externalizing disorders. Regardless of possible contextual differences in CWS across countries [8], the literature is scarce concerning youth in foster care with internalizing symptoms. The scarce literature reflects a lack of attention and awareness. Hopefully, findings from the current study will have implications for how the CWS and health care services meet these youth, with appropriate assessment and interventions.

Multiple risk and protective factors influence the development of internalizing problems (i.e. anxiety and/or depression) in youth. Among these are age [9, 10], sociodemographic factors (e.g. neighborhood stressors and socioeconomic status) [11], biological factors (e.g. genetics, cognitive abilities, physical health) [12, 13] and parental factors (e.g. parental psychopathology and parenting behavior, parent–child interaction) [14, 15]. These various factors may individually or in interaction influence the development of internalizing symptoms [16]. In addition, increased exposure to adverse childhood experiences (e.g. bullying, parental illness, death of family member, divorce, accidents) are found among youth with internalizing disorders [17]. Whereas adverse childhood experiences are frequently reported among youth in the general population [18], youth in foster care have an elevated risk of being exposed to these experiences [19]. Moreover, maltreatment may be specifically relevant to understand the heightened prevalence of internalizing symptoms among youth in foster care.

Maltreatment is defined as *“any act or series of acts of commission or omission by a parent or other caregiver that results in harm, potential for harm, or threat of harm to a child”* [20], although the harm may not be intended. In the present study, we focus on the exposure to maltreatment within the family context, more specifically: physical/emotional abuse, physical/emotional neglect (hereafter neglect) and sexual abuse [21]. Among youth in foster care, a recent study (*N* = 302, mean age 14.8, SD = 2.05) found that 37.0% of the youth reported exposure to emotional or physical abuse; 36.0% reported exposure to neglect, and 24.0% had been exposed to sexual abuse [22].

Previous studies have indicated associations between maltreatment and internalizing problems in adults. Specific types of childhood maltreatment, e.g. sexual abuse, have been associated with increased risk of developing internalizing symptoms in adulthood [23]. In another study, childhood maltreatment was found to predict post-traumatic stress disorder (PTSD) and comorbid internalizing disorders in adults [24]. Nevertheless, while these studies are laudable for addressing an important issue, there are some limitations that challenges the interpretations of the findings. For example, both studies are retrospective studies using adult informants, and they either assessed only one gender, did not differentiate specific populations like high-risk groups, and did not examine subtypes of internalizing problems. Exposure to maltreatment may not necessarily result in a specific type of symptom, i.e. generalized anxiety or depressive symptoms, but it may be related to several different types of internalizing symptoms. Thus, knowledge is limited regarding how specific types of maltreatment associate to specific internalizing symptoms, reported by the youth themselves.

Considering the multiple experiences of maltreatment among youth in foster care, a relevant aspect to investigate is the effect of accumulated exposures of maltreatment on internalizing symptoms. The adverse childhood experiences (ACE) Study reported a cumulative effect of adverse childhood experiences on a broad range of negative health outcomes, including depression, among adults [25]. Similar associations have also been found for other mental disorders [26]. However, retrospective studies increase the risk of recall-bias [27], and responders with internalizing problems have been shown to have a stronger tendency to recall negative life experiences [28].

Furthermore, gender differences have been reported in exposure to different types of maltreatment [29]. It is important to include gender differences when investigating these associations, to increase awareness and enable necessary interventions. Some studies find that girls (aged 12 to 18) more frequently report experiences of physical abuse, neglect and sexual abuse [30]. Other studies find that boys (aged 18 and over) report physical abuse and any other abuse more often [31]. The latter study also found that the association between childhood abuse and internalizing disorders, was stronger for girls than for boys. However, these studies are on selected groups, i.e. juvenile offenders and cross-sectional, and not necessarily transferable to youth in foster care.

One study of children living in foster care (aged 6 to 12 years) show differing results on the association between maltreatment and internalizing disorders, depending on informants, i.e. foster parents and teachers [3]. Exposure to maltreatment and presence of internalizing symptoms may not always be easily identified by caseworkers or caregivers and may explain the discrepancies in research findings. Thus, self-reports from the youth themselves may be highly valuable.

Many factors may contribute to the youth’s mental health while in foster care, with longer time spent in foster care being reported as a protective factor [32, 33]. However, a meta-analysis of 24 studies, investigating developmental outcomes in children in foster care, have not confirmed this. Placements above one year even indicated a worsening in the child’s adaptive functioning, independent of age at placement [34].

The implications of this study may affect how clinicians in the CAMHS and caseworkers in the CWS understand the experiences of youth in foster care, risk factors associated with maltreatment experiences and the development of internalizing symptoms. Levels of internalizing symptoms are generally high among youth in out-of-home care. However, detailed knowledge of symptom profiles of internalizing problems, and their associations with maltreatment types and numbers for youth in foster care is scarce.

### Objectives

In the present study, we examined youth in foster care with regard to: (1) the symptom-levels of subtypes of anxiety and depressive symptoms and the distribution of these symptoms across gender, (2) the rate of exposure to physical/emotional abuse, neglect and sexual abuse for boys and girls respectively, and whether these different forms of maltreatment are differentially associated with subtypes of anxiety and depressive symptoms, (3) whether a cumulative effect is found for increased experiences of maltreatment on depressive and anxiety symptoms, and (4) the potential interaction of gender on exposure to maltreatment and in predicting anxiety and depressive symptoms.

## Method

### Procedure and Participants

This study comprises data from the second wave of the longitudinal cohort study “Young in Foster Care” [35], with data collection completed between October 1, 2016, and March 31, 2017. All youth born between 1999 and 2005, living in foster families within five counties were assessed for eligibility. Eligible youth had lived with their current foster family for a minimum of six months, following legally mandated care by order of the county board.

Through the regional records (*n* = 573) and from the municipal CWS (*n* = 279), a total of 740 youth in foster care were identified as eligible. Another 16 youth were deemed ineligible during the recruitment process, leaving a total of 724 eligible youth. Youth were invited to participate by completing questionnaires, either online on a secure website, or by phone interview. Consistent with the Norwegian legislation, youth aged 11 to 15 years were invited through letters addressed to the foster parents. Youth aged 16 to 18 years were approached directly by postal mail with an invitation and information letter. Youth were compensated with a gift card of 38 USD for their participation.

**Fig. 1:**
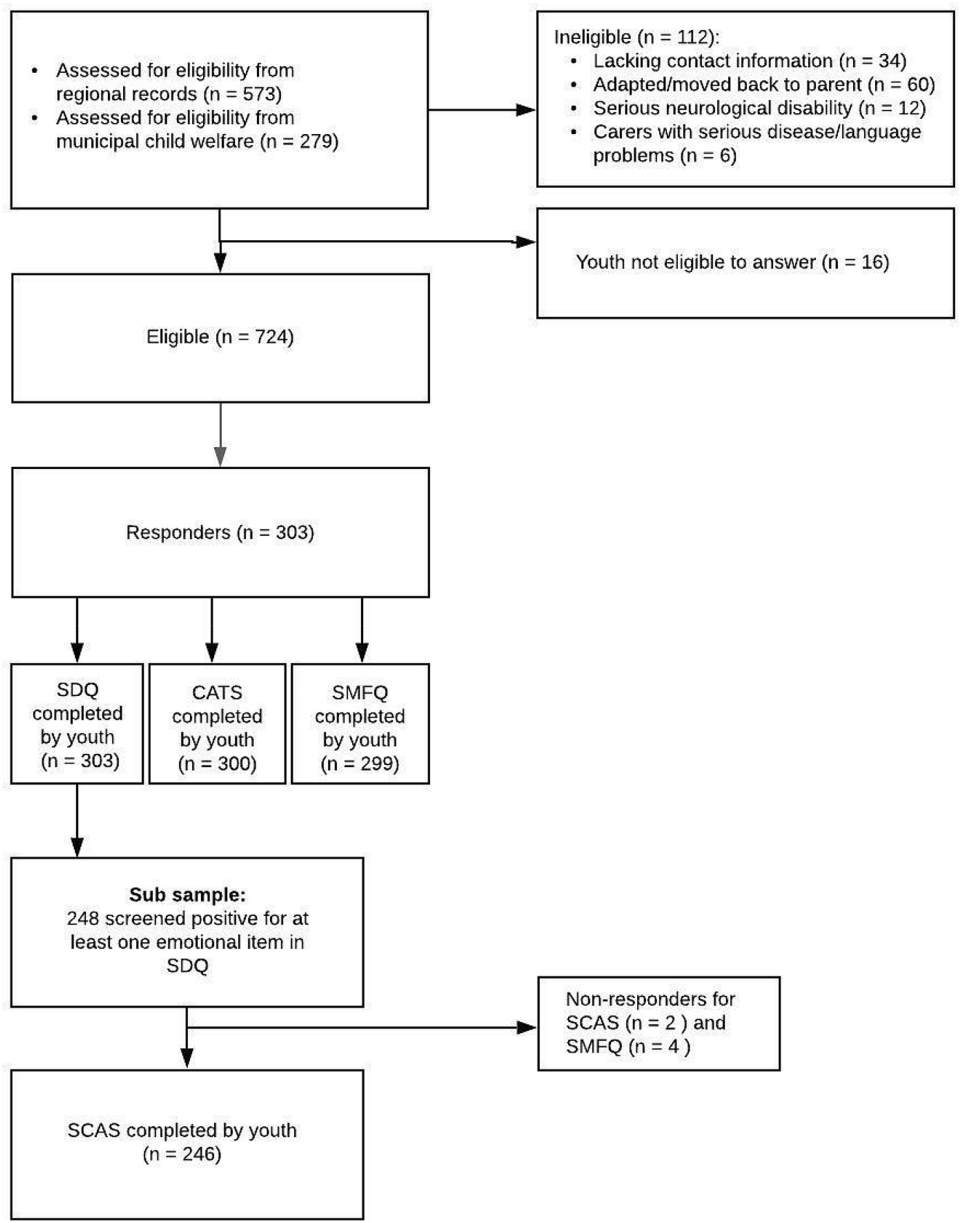
Flowchart of the data-collection

The total sample comprised 303 youth with a response rate of 41.9%. There was an overlap between respondents on the various questionnaires; 303 youth completed the Strengths and Difficulties Questionnaire (SDQ) [36], 299 youth completed the Short Mood and Feelings Questionnaire child version (SMFQ-c) [37], 300 youth completed the Child and Adolescent Trauma Screen youth version (CATS-Y) [38], and 246 youth completed the Spence Children Anxiety Scale child version **(**SCAS-c) [39]. The SDQ was used as a screening of anxiety problems in this study. A total of 248 youth reported problems (“Somewhat true” or “Certainly true”) on at least one item on the SDQ emotional subscale, and hence were asked to complete the SCAS-c. Of these, there were two non-responders, resulting in 246 youth completing SCAS-c. See flowchart, Fig. 1.

Of the total sample, 46.5% were girls, mean age was 14.8 years (SD = 2.04, range 11–18). Mean years living in the current foster family was 6.7 (SD = 4.33, range 0.81–17.63). There were no differences in age and time spent in current foster family between boys and girls. Independent samples *t*-test indicated no significant differences in gender or in time spent in current foster family between responders (*N* = 303) and non-responders (*N* = 421). However, responders were slightly older (*M* = 14.82 years) than non-responders (*M* = 14.30 years, *p* < 0.001).

### Ethics

The Regional Committee for Medical and Health Research Ethics, Western Norway, approved the study. The Norwegian Directorate for Children, Youth and Family Affairs provided exemptions from confidentiality for caseworkers to participate in the study. In accordance with the Norwegian ethics requirement, youth aged 16 to 18 years gave informed consent on their own behalf, while youth aged 12 to 15 years were given the option to participate through consent from their foster parents.

### Measures

#### Sociodemographic Information

Age, gender, and time spent in current foster family was obtained through the regional CWS records of youth in foster care and confirmed by the municipal CWS over telephone.

#### Depression Symptoms

The SMFQ-c [37] was administered to assess depressive symptoms the two previous weeks. The SMFQ-c comprises 13 items rated on a 3-point scale (0 = “Not true”, 1 = “Somewhat true”, 2 = “True”), yielding a total scale score of 26. SMFQ-c has demonstrated good psychometric properties [40–42]. For this study, cut-off for depression problems was set to 11, based on previous studies using same measure [9, 43]. Internal consistency for SMFQ-c in the current sample was excellent (Cronbach’s α = 0.93).

#### Anxiety Symptoms

The SCAS-c [39] was administered to assess anxiety symptoms. The SCAS-c comprises 44 items, including six positive filler items, rated on a 4-point scale (0 = “never” to 3 = “always”) yielding a maximum scale score of 114. The SCAS-c comprise six subscales of anxiety symptoms in line with the following dimensions of anxiety disorders in DSM-IV: Separation anxiety (six items), Social anxiety (six items), Obsessive compulsive disorder (OCD; six items), Panic/Agoraphobia (nine items), Physical injury fears (five items) and Generalized anxiety (six items). SCAS-c has good psychometric properties [44], and has been used in several studies in Norway [45, 46]. Internal consistency for the total scale score of SCAS-c in the current sample was excellent (α = 0.91). Except for the subscale Physical injury fears; α = 0.57, all subscales had acceptable to excellent internal consistency: Separation anxiety; α = 0.70; Social anxiety; α = 0.78; OCD; α = 0.75; Panic/Agoraphobia; α = 0.88; Generalized anxiety; α = 0.88.

#### Maltreatment

We assessed exposure to maltreatment using the youth report version of the CATS-Y for 6 to 18 year-olds, comprising 15 questions on exposure to potential traumatic events [38]. The Norwegian version of CATS have shown good psychometric properties [38]. For this study, we included the six items from CATS-Y covering physical and sexual abuse, and the open-ended item for the youth to indicate exposure to any other experiences not listed. To assess emotional abuse, physical and emotional neglect, we included three items from the ACE Study questionnaire [25]. For assessment of parentification related to neglect, two custom-made items were added. For detailed information about the questionnaire, see [22]. All 10 items were categorized in three main categories: physical and emotional abuse (three items), neglect (four items) and sexual abuse (three items). All items were coded “yes” (= 1) or “no” (= 0). Respondents confirming exposure on at least one item within one category, were coded as 1 on the main category comprising that item. Informants had the option to answer “Pass”. All “Pass” answers were coded as missing. The Cronbach’s Alpha for each main category of maltreatment ranged from acceptable to good: physical and emotional abuse (α = 0.78), neglect (α = 0.73) and sexual abuse (α = 0.68). The lower internal consistency of the maltreatment version in this study is as expected. Different types of maltreatment experiences do not constitute a unified dimension (generating a high internal consistency) but are rather expected to be different experiences occurring concurrently.

#### Statistical Analyses

Statistical analyses were conducted using IBM SPSS Statistics for Windows, Version 25.0. We reported mean scale scores and standard deviations (SD) for SCAS-c and SMFQ-c total scale and SCAS-c subscales for boys and girls separately and collectively. We used independent samples *t*-test to test for possible gender differences. Cohen’s *d* effect sizes were calculated by dividing the group’s mean difference with the pooled standard deviation. Frequencies for the three types of maltreatment are reported for boys and girls separately. Chi Square test and Fisher’s Exact test were used to test for gender differences and Cramer’s V for effect sizes. Effect sizes corresponding to *d* = 0.2 are considered small, *d* = 0.5 as medium and *d* = 0.8 as large [47]. The significance level was set to 0.05. We conducted linear regression analyses where symptoms of depression, total anxiety and anxiety symptoms-subtypes were separately regressed on the independent variables physical/emotional abuse, neglect, and sexual abuse to examine the contribution of each specific subtype of maltreatment. All independent variables were first tested individually, then simultaneously, and finally with age and time spent in current foster family as covariates.

To examine a possible cumulative effect of exposure to several types of maltreatment on symptoms of depression and anxiety we conducted linear regression analysis. The sum score of all types of maltreatment was used as an independent variable and symptoms of depression and anxiety were entered as dependent variables in two separate analyses. The sum score of maltreatment was first tested individually, and secondly adjusted for age and for time spent in current foster family. Possible interaction effects of gender between maltreatment types and internalizing symptoms were examined using multiple regression analysis. All dependent variables were mean centered and standardized prior to entering them into the regression model, enabling comparison of the effect sizes.

#### Missing Data

For SCAS-c, missing responses on item level ranged from 0 to 4.5%. Missing was handled by substituting the missing data with the specific responder’s subscale’s median. To substitute the missing item, the respondent had to have completed 75.0% or more of the items in the scale. For SMFQ-c, missing responses on item level ranged from 0 to 1.0%. For the CATS-Y, missing responses on item level ranged from 0 to 2.0%. The response alternative “pass” varied from 3 to 7.0%. We had information about gender, age and time spent in current foster family, on a total of 292 (96.0%) participants.

## Results

### Symptoms of Depression and Anxiety

Social anxiety (*M* = 5.89, SD = 3.87) and generalized anxiety (*M* = 5.40, SD = 4.21) were the most frequently reported anxiety symptom-subtypes across gender. Girls reported higher scores on all anxiety symptom-subtypes and on depressive symptoms, compared to boys. For depression, 35.3% (*n* = 54) of the girls and 13.8% (*n* = 25) of the boys scored at or above the cut-off score of 11. Effect sizes for gender differences ranged from medium to large [47]. See Table 1 for an overview of internalizing symptoms, distributed by gender.

**Table 1:** Mean and standard deviations of symptoms of anxiety and depression for boys and girls separately, with independent *t*-tests for gender differences

	*n*	Range	Mean	SD	t	df	*p*	ES
SCAS-c (total scale score)					−7.229	243	p < 0.001	0.93
Boys	118	54	17.97	11.90				
Girls	127	89	33.41	20.19				
SCAS-c subscale scores								
Separation anxiety					−4.986	243	p < 0.001	0.64
Boys		10	2.06	2.31				
Girls		13	3.87	3.26				
Social anxiety					−6.081	243	p < 0.001	0.78
Boys		13	4.44	3.13				
Girls		17	7.25	4.01				
Obsessive compulsive disorder					−4.546	243	p < 0.001	0.59
Boys		13	2.82	2.66				
Girls		18	4.69	3.64				
Panic/agoraphobia					−5.941	243	p < 0.001	0.77
Boys		18	2.09	2.97				
Girls		24	5.70	5.86				
Physical injury fears					−6.042	243	p < 0.001	0.77
Boys		10	2.90	2.30				
Girls		13	4.92	2.89				
Generalized anxiety					−6.781	243	p < 0.001	0.88
Boys		16	3.65	2.84				
Girls		18	7.02	4.64				
SMFQ-c (total scale score)					−5.988	296	p < 0.001	0.69
Boys	159	22	5.03	5.02				
Girls	139	26	9.68	8.19				

*Note*. Symptoms of anxiety was measured with Spence Children’s Anxiety Scale Child (SCAS-c); Symptoms of depression was measured with The Short Mood and Feelings Questionnaire Child (SMFQ-c). *ES* = effect size, calculated as Cohen’s *d*

### Frequency of Maltreatment Distributed by Gender

Maltreatment was reported by 58.3% of the girls and 35.2% of the boys. Girls reported exposure to more types of maltreatment (*M* = 2.16, SD = 2.52) than boys (*M* = 1.06, SD = 1.84, *t* = − 4.25, *p* < 0.001). Effect sizes for gender differences ranged from small to medium. See Table 2 for the frequency of exposure to physical/emotional abuse, neglect, and sexual abuse, distributed by gender.

**Table 2:** Frequency of maltreatment reported by youth in foster care, for boys and girls separately

Have you experienced	*N*	Yes (n)	%	*p*	ES
Physical/emotional abuse	296	94	31.8	0.016	0.19
Boys	158	38	24.1		
Girls	138	56	40.6		
Neglect	298	107	35.9	0.004	0.22
Boys	159	42	26.4		
Girls	139	65	46.8		
Sexual abuse	295	50	16.9	< 0.001	0.24
Boys	156	14	9.0		
Girls	139	36	25.9		

*Note*. Chi-square test. Fisher’s exact is applied for Neglect and Sexual abuse. P-value reported for significant differences between the genders. *ES* = effect size, calculated as Cramer’s V

### Associations Between Maltreatment Types and Depressive Symptoms

There were significant main effects (*p* < 0.001) for all three types of maltreatment on symptoms of depression (Table 3). The association between physical/emotional abuse and depressive symptoms was no longer significant when adjusted for neglect and sexual abuse. In the fully adjusted model, older age (*β* = 0.66, *p* < 0.001) was associated with having more depressive symptoms. Overall, the fully adjusted model explained 28.9% of the total variance in depressive symptoms. The explained variance increased significantly from the unadjusted to the adjusted model, and further to the fully adjusted model, adding age and time spent in current foster family as covariates.

**Table 3:** Associations between types of maltreatment and symptoms of depression (*n* = 282) and anxiety (*n* = 231) among youth in foster care

	Symptoms of depression			Symptoms of anxiety		
	*b* (S.E)	ES	*R*^2^	*b* (S.E)	ES	*R*^2^
Unadjusted						
Physical/emotional abuse	**2.53(0.39)**	0.36	0.13	**7.15(1.07)**	0.39	0.16
Neglect	**2.49(0.34)**	0.35	0.16	**7.00(0.97)**	0.38	0.19
Sexual abuse	**4.50(0.59)**	0.64	0.17	**11.30(1.63)**	0.62	0.17
Adjusted			0.25			0.28
Physical/emotional abuse	0.74(0.50)	0.11		2.64(1.36)	0.14	
Neglect	**1.41(0.44)**	0.20		**3.80(1.24)**	0.21	
Sexual abuse	**3.26(0.61)**	0.46		**7.61(1.67)**	0.42	
Full adjusted model			0.29			0.28
Physical/emotional abuse	0.74(0.48)	0.10		2.66(1.36)	0.15	
Neglect	**1.27(0.44)**	0.18		**3.92(1.28)**	0.21	
Sexual abuse	**2.90(0.61)**	0.41		**7.67(1.69)**	0.42	
Age	**0.66(0.18)**	0.09		−0.15(0.54)	−0.01	
Time spent in current foster family	0.01(0.09)	0.00		0.09(0.25)	0.01	

*Note*. Dependent variables: Symptoms of depression was measured with The Short Mood and Feelings Questionnaire Child; Symptoms of anxiety was measured with Spence Children’s Anxiety Scale Child. Predictors: *Unadjusted analyses* = physical/emotional abuse, neglect, and sexual abuse are tested separately. *Adjusted analyses* = physical/emotional abuse, neglect and sexual abuse are added simultaneously. *Full adjusted model* = physical/emotional abuse, neglect, sexual abuse, age, and time spent in current foster family added simultaneously. *b* = unstandardized coefficient of the predictor; *S.E* = Standard error of the coefficient. *ES* = effect size, standardized z-score; *R*^2^ = R Squared. The statistically significant results are marked in boldface

### Associations Between Maltreatment Types and Anxiety Symptoms

There were significant main effects (*p* < 0.001) for all three types of maltreatment on total symptoms of anxiety (Table 3). In the fully adjusted model, the strongest association was found for sexual abuse (*β* = 7.67, *p* < 0.001). Also, here, the association between physical/emotional abuse and symptoms of anxiety was no longer significant when adjusting for neglect and sexual abuse. Overall, the fully adjusted model explained 27.8% of the total variance in anxiety symptoms. The explained variance increased significantly from the unadjusted to the adjusted model but did not increase when adding age and time in current foster family as covariates in the fully adjusted model.

Furthermore, sexual abuse was associated with all anxiety symptom-subtypes in the fully adjusted models with small to medium effect sizes, from 0.28 (social anxiety) to 0.39 (OCD). Neglect was associated with all anxiety symptom-subtypes with small effect sizes, from 0.17 (social anxiety) to 0.29 (panic/agoraphobia), except separation anxiety and physical injury fears. Physical/emotional abuse was associated only with social anxiety and OCD, with small effect sizes (0.17 and 0.18, respectively). See Table 4.

**Table 4:** Associations between types of maltreatment and anxiety symptom-subtypes among youth in foster care (*N* = 231)

	Separation Anxiety			Social anxiety			OCD			Panic/agoraphobia			Physical Injury Fears			Generalized anxiety		
	*b* (S.E)	ES	*R*^2^	*b* (S.E)	ES	*R*^2^	*b* (S.E)	ES	*R*^2^	*b* (S.E)	ES	*R*^2^	*b* (S.E)	ES	*R*^2^	*b* (S.E)	ES	*R*^2^
Unadjusted																		
Physical/emotional abuse	**0.61(0.19)**	0.21	0.05	**1.46(0.23)**	0.38	0.15	**1.26(0.20)**	0.38	0.15	**1.81(0.30)**	0.36	0.14	**0.50(0.18)**	0.18	0.03	**1.51(0.25)**	0.36	0.14
Neglect	**0.51(0.17)**	0.17	0.04	**1.35(0.21)**	0.35	0.16	**1.16(0.18)**	0.35	0.15	**2.01(0.26)**	0.40	0.21	**0.32(0.16)**	0.12	0.02	**1.65(0.22)**	0.39	0.19
Sexual abuse	**1.22(0.28)**	0.41	0.08	**1.97(0.36)**	0.51	0.12	**1.86(0.31)**	0.56	0.14	**2.82(0.45)**	0.56	0.15	**1.00(0.27)**	0.36	0.06	**2.43(0.39)**	0.58	0.15
Adjusted			0.09			0.22			0.23			0.27			0.07			0.26
Physical/emotional abuse	0.29(0.25)	0.10		**0.69(0.30)**	0.18		**0.59(0.26)**	0.18		0.39(0.37)	0.08		0.35(0.24)	0.12		0.34(0.32)	0.08	
Neglect	0.13(0.23)	0.04		**0.68(0.27)**	0.18		**0.55(0.23)**	0.16		**1.40(0.34)**	0.28		−0.07(0.22)	−0.02		**1.12(0.29)**	0.27	
Sexual abuse	**0.99(0.30)**	0.33		**1.19(0.37)**	0.31		**1.21(0.32)**	0.36		1.78(0.46)	0.36		**0.86(0.29)**	0.31		**1.58(0.39)**	0.38	
Full adjusted model			0.12			0.24			0.24			0.27			0.07			0.26
Physical/emotional abuse	0.32(0.25)	0.11		**0.66(0.30)**	0.17		**0.61(0.26)**	0.18		0.39(0.38)	0.08		0.35(0.24)	0.13		0.33(0.32)	0.08	
Neglect	0.18(0.23)	0.06		**0.65(0.28)**	0.17		**0.60(0.24)**	0.18		**1.43(0.35)**	0.29		−0.06(0.22)	−0.02		**1.12(0.30)**	0.27	
Sexual abuse	**1.10(0.30)**	0.37		**1.07(0.37)**	0.28		**1.30(0.32)**	0.39		**1.77(0.47)**	0.35		**0.87(0.30)**	0.31		**1.54(0.40)**	0.37	
Age	−**0.23(0.01)**	−0.08		0.22(0.12)	0.06		−0.18(0.10)	−0.06		0.01(0.15)	0.00		−0.03(0.09)	−0.01		0.07(0.13)	0.02	
Time spent in current foster family	0.01(0.05)	0.00		0.01(0.06)	0.00		0.02(0.05)	0.01		0.03(0.07)	0.01		0.00(0.04)	0.00		0.02(0.06)	0.01	

*Note*. Dependent variables: Symptoms of depression was measured with The Short Mood and Feelings Questionnaire Child; Symptoms of anxiety was measured with Spence Children’s Anxiety Scale Child. Separation anxiety, social anxiety, OCD, panic/agoraphobia, physical injury fears, generalized anxiety are subscales part of SCAS-c. Predictors: *Unadjusted analyses* = physical/emotional abuse, neglect, and sexual abuse are tested separately. *Adjusted analyses* = abuse, neglect and sexual abuse are added simultaneously. *Full adjusted model* = physical/emotional abuse, neglect, sexual abuse, age, and time spent in current foster family added simultaneously. *b* = unstandardized coefficient of the predictor; *S.E* = Standard error of the coefficient; *ES* = effect size, standardized z-score; *R*^2^ = *R* Squared. The statistically significant results are marked in boldface

### Cumulative Effect of Number of Maltreatment Types

Higher numbers of maltreatment types were associated with more symptoms of depression and anxiety. The association remained significant when controlling for age and time in current foster family. Increased age was associated with more symptoms of depression. See Table 5.

**Table 5:** Cumulative effect of maltreatment on symptoms of depression (*n* = 286) and anxiety (*n* = 235)

	Symptoms of depression			Symptoms of anxiety		
	*b* (S.E)	ES	*R*^2^	*b* (S.E)	ES	*R*^2^
Unadjusted			0.23			0.27
Maltreatment sum score	**1.46(0.16)**	0.21		**4.06(0.44)**	0.22	
Adjusted			0.27			0.27
Maltreatment sum score	**1.34(0.17)**	0.19		**4.12(0.48)**	0.23	
Age	**0.70(0.18)**	0.10		−0.05(0.53)	−0.00	
Time spent in current foster family	0.03(0.09)	0.00		0.11(0.25)	0.01	

*Note.* Dependent variables: Symptoms of depression was measured with The Short Mood and Feelings Questionnaire Child; Symptoms of anxiety was measured with Spence Children’s Anxiety Scale Child. Predictors: *Unadjusted analyses* = maltreatment sum score is tested separately. *Adjusted analyses* = maltreatment sum score, Age and Time spent in current foster family added simultaneously. *b* = unstandardized coefficient of the predictor; *S.E* = Standard error of the coefficient; *ES* = effect size, standardized z−score; *R*^2^ = *R* Squared. The statistically significant results are marked in boldface

### Interaction Effects of Gender

Significant interaction effects were found between gender and all three types of maltreatment on symptoms of anxiety, where the associations were stronger for girls compared to boys (physical/emotional abuse; *β* = 0.63, *F*(3, 238) = 39.80, *p* < 0.001, neglect; *β* = 0.48, *F*(3, 240) = 38.71, *p* = 0.019, sexual abuse; *β* = 0.57, *F*(3, 237) = 35.58, *p* = 0.041). Regarding symptoms of depression, there was a significant interaction effect between gender and neglect, where the associations were stronger for girls compared to boys; *β* = 0.44, *F*(3, 292) = 31.02, *p* = 0.016.

## Discussion

The aim of this study was to examine patterns of internalizing symptoms among youth in foster care, and to investigate whether types and numbers of maltreatment were differentially associated with types of internalizing symptoms. The results indicated higher internalizing symptom-levels in girls relative to boys. Mean levels of symptoms of depression and anxiety were generally higher compared to that of other youth community samples using same measures and cut-off score [44, 48, 49]. However, mean levels were below clinical cut-off on all symptoms. All three types of maltreatment were associated with internalizing symptoms, with the strongest associations found for sexual abuse, followed by neglect. A cumulative effect was found between maltreatment and internalizing symptoms. Overall, the associations between all maltreatment types and anxiety symptoms, and between neglect and depressive symptoms were stronger for girls than for boys.

### Prevalence of Internalizing Symptoms Among Boys and Girls in Foster Care

Our results are in accordance with previous findings demonstrating a high prevalence of internalizing symptoms among children and youth in foster care [1, 50]. Youth in the present sample scored significantly higher on the anxiety total scale and most symptom-subtypes, compared to both a Danish community sample of 12 to 17 year-olds (*N* = 345) [44], and a Dutch community sample of 12 to 18 year-olds (*N* = 968) [49].

Contrary to our findings, a previous study of younger children in foster care found no gender differences regarding internalizing problems [3]. This discrepancy may be explained by a general gender difference in internalizing symptoms among adolescents, but not among children. Studies on both general and clinical youth populations have found gender differences in prevalence of internalizing problems with increasing age [51, 52]. Our findings indicate that youth in foster care also follow this developmental trajectory and seem to be comparable to their peers in this respect. Although gender differences are not apparent during childhood, the clinical implications of the change during adolescence, points to the need for preventive measures during childhood and adolescents for girls.

Social anxiety and generalized anxiety were the highest symptom-subtypes among youth in this study. Consistent with other youth populations, social anxiety and generalized anxiety gets more prominent in adolescence [53]. Despite the youth’s increased risk of exposure to maltreatment and higher levels of symptoms, one may hypothesize that youth in foster care are more similar than different from their peers regarding which types of anxiety that are most prevalent within the groups. Though, in this study, we do not have adequate data to explore this further.

Regarding depressive symptoms, the mean total symptom score (*M* = 7.20, SD = 7.06) was comparable to the level of depressive symptoms found among internationally adopted youth aged 16–19 in Norway (*N* = 45, *M* = 8.34, SD = 7.59) [54]. In a study of Norwegian adolescents in the general population aged 10–19 (*N* = 5804) [48] using same depression measure and cut-off, the total mean symptom score is significantly lower compared to the present foster youth sample (*M* = 4.50 SD = 4.72, *t* = 9.37, *df* = 6101, *p* < 0.001). In the foster youth sample 23.7% scored at or above cut-off, indicating a higher proportion of clinically significant symptom levels than the general adolescent population with 11.2% scoring above cut-off [48]. The higher percentage in the foster care sample may be explained by various factors, including the foster care placement(s), inadequate access to mental health services and their history of maltreatment. To summarize, youth in foster care have heightened levels of internalizing symptoms relative to other youth community samples. However, they may seem to follow the same development in terms of patterns of internalizing symptoms most prevalent from childhood to youth.

### Maltreatment and Associations with Internalizing Symptoms

Boys reported lower frequencies of all three types of maltreatment compared to girls. Similar findings were reported in a systematic review on the prevalence of self-reported child maltreatment, where varying prevalence was found depending on type of maltreatment, across continents and gender [55]. Furthermore, our findings are in line with studies showing that girls more frequently report having been exposed to sexual abuse compared to boys generally [56]. This may be due to actual differences in exposure. However, underreporting of sexual abuse by boys may be caused by other reasons, such as stigma, shame and myths of boys being less likely to be exposed to sexual abuse. Boys may also be more subjected to blame for provoking the sexual abuse or afraid of being perceived as weak or less masculine by their peers [57, 58].

Maltreatment was associated with internalizing symptoms, which is consistent with other research suggesting that early experiences of family violence links to undesirable health outcomes later in life, including depression and anxiety [59]. We also found specificity of maltreatment which is in line with a meta-analysis investigating the role of specific, early experiences of trauma in adult depression [60]. In this meta-analysis, depression was most strongly associated with emotional abuse, followed by neglect and sexual abuse, and to a lesser extent physical abuse. Our results indicate that sexual abuse and neglect have a stronger impact, in terms of being associated with a larger range of internalizing symptoms, compared to that of physical abuse. This may be related to the finding that physical abuse affects anger-dysregulation [61], whereas sexual abuse and neglect may enhance feelings of worth- and powerlessness, shame and guilt [62] and thus, more related to the development of internalizing symptoms.

Contrary to our findings, a study of younger children in foster care, found no associations between neglect and internalizing symptoms [3]. This inconsistency may partly be explained by the use of self-report of internalizing symptoms in the present study, and a more detailed assessment of self-reported neglect and abuse compared to the previous study.

Our findings indicate that self-reports on maltreatment experiences may have relevance in research, and probably also in clinical settings. Often, case workers in the CWS seek information from other informants [63] which is valuable, but do not necessarily reflect the youth’s subjective experience. Also, internalizing symptoms, particularly in adolescents, may be difficult for caregivers and others to recognize. Furthermore, our findings also suggest the importance of using standardized assessment tools, covering a wide range of experiences of maltreatment, as well as symptoms experienced by the youth.

The associations between maltreatment experiences and internalizing symptoms in this study are substantial, mostly with medium-to-large effect sizes. One plausible explanation may be the high prevalence rates of both maltreatment experiences and symptoms, accentuating the effect sizes. Considering these youth’s background and risk of mental health problems, the strength of these associations is not surprising. Furthermore, considering the consistency of our findings, the results carry clinical implications, in highlighting the importance of focusing on both the youth’s experiences and present symptoms. These findings communicate clearly to the youth and their caregivers how previous maltreatment experiences often go hand in hand with present mental health issues.

### Cumulative Effect of Maltreatment on Internalizing Symptoms

Increased number of maltreatment experiences was associated with higher levels of internalizing symptoms, confirming previous findings in adult samples [64]. Highlighting a cumulative effect of maltreatment may nuance the perspective of specificity of *types* of maltreatment as risk factors for internalizing problems. Our findings support that the number of maltreatment experiences, regardless of type, is relevant for the levels of internalizing symptoms. Taken together, our findings indicate that it may be helpful to emphasize both a cumulative effect and type of exposure to maltreatment. They each contribute to enhanced understanding of the risk for internalizing symptoms among youth in foster care, and thus, may generate a more comprehensive understanding.

### Interaction Effects of Gender

The association between all types of maltreatment and symptoms of anxiety was stronger for girls. Regarding depression, an interaction effect of gender was found only for girls who had been exposed to neglect. Overall, the interaction effect of gender was greater on symptoms of anxiety compared to depressive symptoms, when exposed to maltreatment. Some researchers hypothesize that girls may be more vulnerable when experiencing stressors, and thus, more receptive for difficulties in stressful situations, compared to boys [65]. Researchers have found that experiences of sexual abuse in childhood predicted earlier, perceived pubertal development for girls. This may in turn be associated with increased internalizing symptoms [66]. However, one study found gender differences in the expression of internalizing versus externalizing symptoms when exposed to distinct types of maltreatment [67]. Physical abuse was associated with externalizing symptoms in men, and internalizing symptoms in women. Thus, girls and boys may respond contrarily when exposed to maltreatment, and our results may reflect this.

### Strengths and Limitations

The study is among few focusing on internalizing symptoms and maltreatment in a foster care population using self-reports, addressing an expressed need [68]. Among the strengths of the study is the inclusion of youth in foster care from several counties and the use of validated instruments to measure both exposure to maltreatment and internalizing symptoms. Furthermore, the timespan between exposure and reporting was limited compared to retrospective studies with adult informants on childhood adversities. The use of self-reported internalizing symptoms is important, considering the difficulty of obtaining valid responses on internalizing symptoms from caregivers [69].

Although there are strengths using self-report data, such as its availability and cost efficiency, the limitations of self-reports may bias results in terms of social desirability, the youth’s lack of introspection or the context when reporting [70]. Ideally, multi-informant perspectives should be used [71], e.g. with reports from foster parents, added to the youth’s self-reports. However, considering the lack of studies with youth self-reports in this population, this will generate a valuable contribution in this field.

The response rate (41.9%), although smaller than we aimed for, represents a relative high proportion of a population that is considered hard to recruit. Furthermore, no differences were found between responders and non-responders regarding gender and time spent in current foster family, suggesting that the sample is unbiased on important variables. However, compared to non-responders, responders were older. Older youth may be more aware of their situation and experiences, and thus, more likely to respond or find the questions comprehensible. As the experiences of younger foster youth are not represented to the same extent as the older, our findings may be more representative for older foster youth.

Furthermore, the lack of in-depth data on maltreatment may leave us less informed on the potential extent of harm stated in the youth’s reports. For example, regarding sexual abuse, we do not know the youth’s relation to the perpetrator, the duration, age, or timing of the abuse. It is possible that experiences of maltreatment may generate different outcomes depending on when in life they occur [72].

Furthermore, to minimize the burden of completing SCAS-c when anxiety symptoms were not relevant, the SDQ was used as a screening for anxiety problems. This may have resulted in missing some participants in the more thorough assessment of anxiety symptoms. However, SDQ is considered to have acceptable screening properties for this population [4]. Lastly, the cross-sectional design of this study does not allow to draw causal relationships between exposure to maltreatment and internalizing symptoms. Thus, we can only report our findings in terms of associations.

## Summary

In the present study, youth in foster care reported generally higher mean levels of internalizing symptoms, with more youth scoring above cut-off compared to youth in community samples. We found associations and specificity between maltreatment and a broad range of internalizing symptoms, particularly for sexual abuse and neglect. Furthermore, compared to boys, girls reported higher levels of anxiety symptoms when exposed to all types of maltreatment, and higher levels of depressive symptoms when exposed to neglect. Both type and cumulative effect of maltreatment are risk factors to consider in clinical work with children and youth in foster care.

The clinical implications of this study are relevant for clinicians in mental health services who meet youth from foster homes in clinical settings. Also, caseworkers in CWS could benefit from this knowledge, being recommended to assess a broad range of different types of maltreatment experiences, as well as subtypes of internalizing symptoms among youth in foster care. Increased knowledge about internalizing symptoms and their associations with maltreatment is important to understand the needs of these youth. It can also help navigate the direction for further referrals, and thereby have an impact on the youth’s access to services in accordance with their needs.

This study represents an important empirical contribution by emphasizing a holistic perspective on youth in foster care. Clinicians should not have a one-sided focus on the youths’ history as opposed to their symptoms and impairments, but rather have an understanding of the associations between their history and their mental health symptoms. Hence, the youth’s life story should be validated, as well as acknowledging that their potential traumatic experiences carries risks for mental health symptoms.

The dearth of studies focusing on internalizing symptoms among youth in foster care, indicate the need for more research to replicate our findings. Future studies are recommended to include effect sizes to enable comparisons of results. The inclusion of multiple informants, e.g., foster parents, and inclusion of longitudinal data to investigate the developmental trajectories of youth placed in foster care, would be highly valuable. Recognizing different types of maltreatment experiences found among youth in foster care, one may speculate whether they will benefit from treatment interventions for internalizing symptoms to the same degree as their peers. Future research should provide insights into whether patterns, type, and level of internalizing symptoms in youth in foster care are similar and/or different compared to youth in general populations and/or clinical samples.
